# Comparative Evaluation of Wear of Natural Teeth and Crown Materials Opposing Metal, Metal-Ceramic, and Zirconia Surfaces: An In Vitro Study

**DOI:** 10.7759/cureus.105571

**Published:** 2026-03-20

**Authors:** Chamanthi P, CN Anusha Naik, Lokanathan Balaji Doddy, Gayathri M, Navreen Kaur, Jhansi Siliveri

**Affiliations:** 1 Prosthodontics, Chadalawada Krishna Srinivasa Teja Institute of Dental Sciences and Research, Tirupati, IND; 2 Prosthodontics, AIIMS (All India Institute of Medical Sciences), Raebareli, Raebareli, IND; 3 Prosthodontics, Priyadarshini Dental College and Hospital, Tiruvallur, IND; 4 Pedodontics and Preventive Dentistry, G. Pulla Reddy Dental College and Hospital, Kurnool, IND; 5 Dentistry, Sri Guru Ram Das Institute of Dental Sciences and Research, Amritsar, IND

**Keywords:** dental crowns, dental enamel, dental porcelain, tooth wear, zirconium oxide

## Abstract

Background

The increasing demand for aesthetic dentistry has led to widespread use of zirconia and metal-ceramic restorations. Although zirconia demonstrates superior mechanical properties and fracture resistance, the wear behaviour of restorative materials against natural enamel and opposing restorations remains a critical clinical consideration.

Objective

To comparatively evaluate the surface and volumetric wear of natural teeth, metal-ceramic crowns, and zirconia crowns when opposed by metal, metal-ceramic, and zirconia surfaces under standardised in vitro conditions.

Materials and methods

Ninety freshly extracted human premolars were randomly allocated into three groups (n = 30): natural teeth, metal-ceramic crowns, and zirconia crowns. Each group was further subdivided (n = 10) according to opposing disc material (metal, metal-ceramic, or zirconia). Specimens were mounted in acrylic resin and subjected to wear testing using a pin-on-disc apparatus under a constant 2 kg load for 10 cycles of one-hour duration with continuous distilled water irrigation at 37°C. Surface and volumetric wear were measured after testing. Statistical analysis was performed using one-way analysis of variance (ANOVA) with a significance level set at p < 0.05.

Results

Significant differences in both surface and volumetric wear were observed among groups (p < 0.05). Metal-ceramic antagonists produced the highest surface wear across natural teeth (177.2 ± 40.4), metal-ceramic crowns (181.3 ± 37.6), and zirconia crowns (141.4 ± 56.7). Metal discs demonstrated the lowest surface wear in all categories. Volumetric analysis revealed that zirconia opposing surfaces consistently resulted in the least material loss.

Conclusions

Wear behaviour varied significantly depending on restorative and opposing material combinations. Metal-ceramic antagonists generated the greatest wear, whereas zirconia surfaces demonstrated comparatively favourable and enamel-compatible wear characteristics. Polished zirconia may represent a clinically acceptable balance between aesthetics and functional durability.

## Introduction

The growing emphasis on aesthetic dentistry has significantly increased the use of tooth-coloured restorative materials in prosthodontic treatment. All-ceramic crown systems have gained popularity because of their superior aesthetic appearance and biocompatibility when compared with traditional metal-ceramic restorations [1]. Earlier ceramic materials were limited by brittleness and insufficient tensile strength; however, the development of reinforced ceramics containing aluminium oxide, leucite, lithium disilicate, and zirconia has markedly improved their mechanical properties [2]. Zirconia, in particular, demonstrates exceptional structural stability due to its polymorphic crystalline structure and transformation-toughening mechanism, which enhances resistance to crack propagation. These characteristics have expanded its clinical application in both single-unit crowns and fixed partial dentures [3].

Wear of dental structures is a continuous biological phenomenon resulting from repeated contact and sliding between opposing surfaces. This process may be accelerated when restorative materials exhibit wear characteristics that differ from those of natural enamel. Excessive enamel wear can lead to significant clinical consequences, including occlusal surface deterioration, reduction in vertical dimension, compromised masticatory efficiency, dentin hypersensitivity, and aesthetic concerns [4]. Multiple factors within the oral environment contribute to wear, including attrition from occlusal contact, abrasion from dietary and oral hygiene factors, and corrosion caused by chemical exposure such as acidic foods and gastric reflux [5].

Previous investigations have indicated that ceramic restorative materials may produce greater antagonistic enamel wear compared with other restorative options. The wear performance of ceramics is influenced by several interrelated variables, including surface roughness, microstructure, hardness, fracture toughness, loading conditions, and environmental factors [4]. Surface finishing techniques are particularly important, as polished ceramic surfaces have been associated with reduced enamel wear compared with glazed surfaces. Furthermore, ceramics typically undergo wear through microfracture mechanisms, which differ from the adhesive wear patterns observed in metals and composite resins [6].

An ideal restorative material should demonstrate wear behaviour compatible with natural enamel to maintain long-term occlusal harmony. Although earlier ceramic systems showed inferior mechanical properties relative to metal-ceramic restorations, modern zirconia-based materials exhibit improved fracture resistance and durability due to their phase transformation characteristics [3]. While clinical studies provide valuable information regarding restorative wear, they are often limited by variability in patient-specific factors and the high cost and duration of long-term observation. In contrast, in vitro experimental models enable standardised assessment of wear by controlling critical variables such as load, motion, and environmental conditions. Despite inherent limitations, laboratory simulations remain an effective method for comparative evaluation of restorative materials [7].

In view of the increasing clinical use of zirconia and metal-ceramic restorations, a systematic in vitro assessment of their wear behaviour against natural teeth and opposing restorative surfaces is essential to guide evidence-based material selection. The present in vitro study aimed to comparatively evaluate the wear behaviour of natural teeth, metal-ceramic crowns, and zirconia crowns when opposed by metal, metal-ceramic, and zirconia surfaces under standardised laboratory conditions.

## Materials and methods

Study design and sample selection

**Figure 1:**
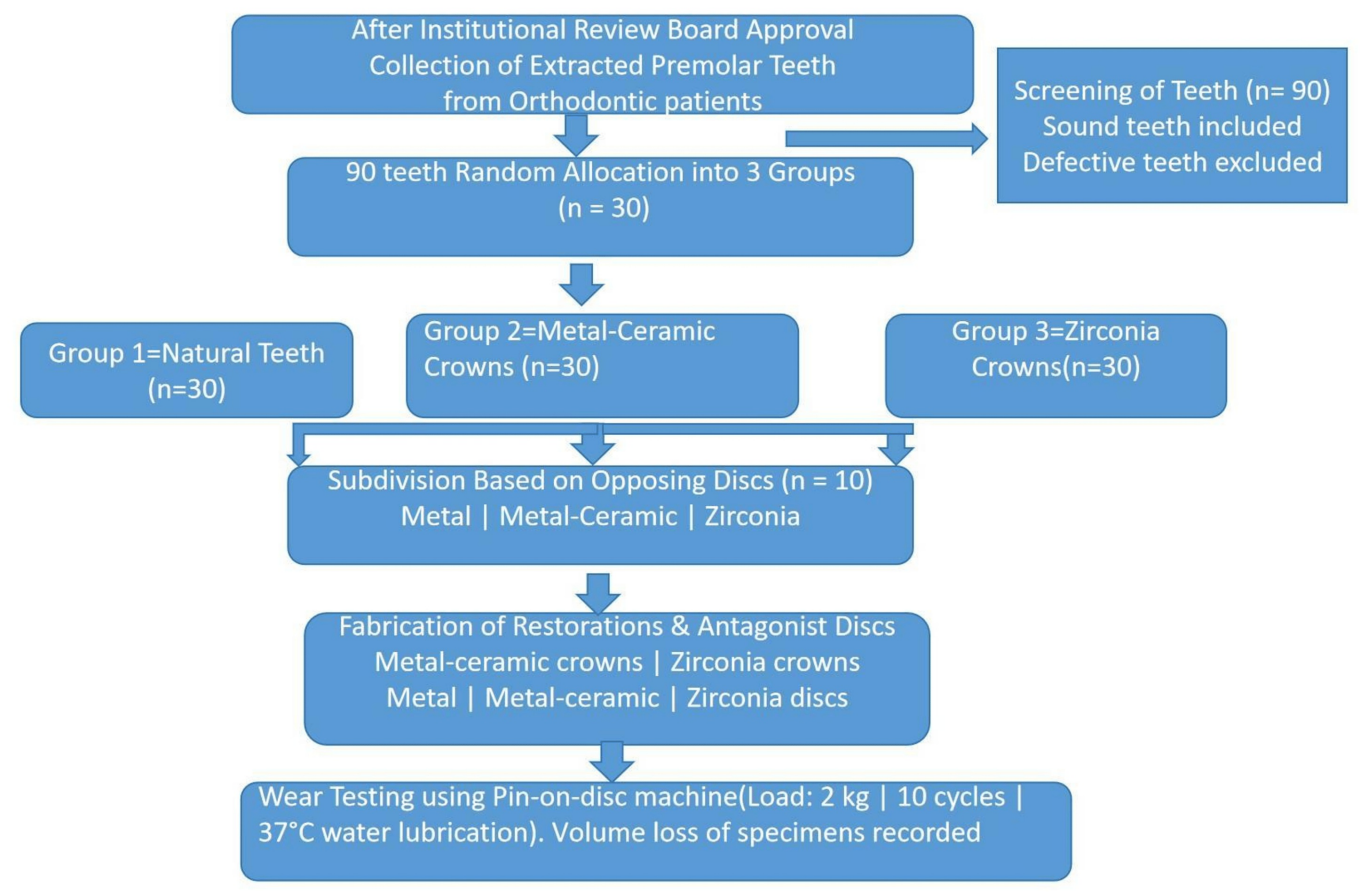
Study protocol

**Figure 2:**
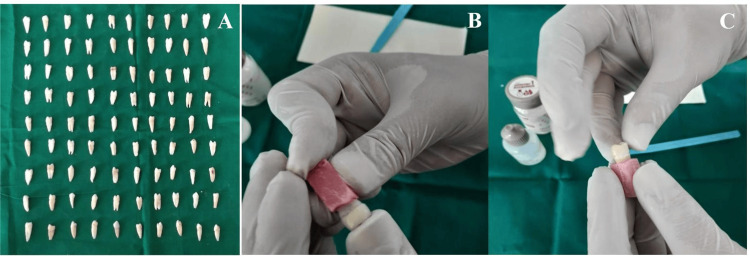
Samples used in the study A) Samples of orthodontic extracted human natural premolars. B) Cemented metal-ceramic crown. C) Cemented zirconium crown.

This in vitro experimental study was conducted using freshly extracted human premolar teeth obtained from orthodontic patients aged 13-30 years after informed consent and institutional approval. The overall study workflow and experimental protocol are illustrated in Figure 1. The sample size was calculated using G*Power software (version 3.1, Heinrich Heine University, Düsseldorf, Germany) for one-way analysis of variance (ANOVA) involving three groups. Based on previous similar in vitro wear studies, a medium effect size (f = 0.40) was assumed [3,6]. With a significance level (α) of 0.05 and statistical power of 80%, the minimum required sample size was determined to be 84 specimens (28 per group). To compensate for potential specimen loss during fabrication or testing, 90 specimens were included, with 30 allocated to each group (Figure 2A).

Ninety sound premolar teeth free from caries, cracks, restorations, or structural defects were selected. The teeth were cleaned of debris and stored in sterile distilled water at 37°C until testing to prevent dehydration. The specimens were randomly allocated into three groups of 30 each: natural teeth, metal-ceramic crowns, and zirconia crowns. Each group was further subdivided into three subgroups (n = 10) according to the opposing disc material (metal, metal-ceramic, or zirconia). All specimens were embedded in auto-polymerising acrylic resin blocks (2 cm × 1 cm) to ensure standardized mounting in the wear testing apparatus.

Fabrication of restorative samples

For the metal-ceramic group, standardized tooth preparation was performed using a high-speed handpiece with diamond burs, providing approximately 1.5 mm occlusal reduction and a shoulder finish line [8]. Impressions were made using polyvinyl siloxane material with a two-stage putty-wash technique and poured using type IV die stone. Wax patterns were fabricated and invested in phosphate-bonded investment material, and casting was carried out using a nickel-chromium alloy. Feldspathic porcelain was layered over the metal framework using opaque and dentin porcelain and fired in a ceramic furnace according to the manufacturer’s instructions, followed by glazing. After glazing, the crown surfaces were finished and polished using a sequential polishing protocol with fine and superfine diamond burs, followed by silicon carbide abrasive discs of increasing grit sizes (600-1,200 grit) under water irrigation. Final polishing was performed using diamond polishing paste with a felt wheel for approximately 60 seconds to obtain a smooth and standardized surface prior to wear testing [9]. The finished crowns were cemented onto prepared teeth using glass ionomer cement under standardized seating pressure (Figure 2B).

For the zirconia group, prepared teeth were scanned using a three-dimensional laboratory scanner. Crowns with standardized morphology were designed using computer-aided design (CAD) software (Exocad GmbH, Darmstadt, Germany) and milled from pre-sintered 3 mol% yttria-stabilized tetragonal zirconia polycrystal (3Y-TZP) zirconia blocks (Ivoclar Vivadent AG, Schaan, Liechtenstein) using a five-axis milling unit. Sintering was completed according to the manufacturer’s specifications [10,11]. Cementation was performed using the same protocol as in the metal-ceramic group to maintain consistency (Figure 2C).

Fabrication of opposing discs

**Figure 3:**
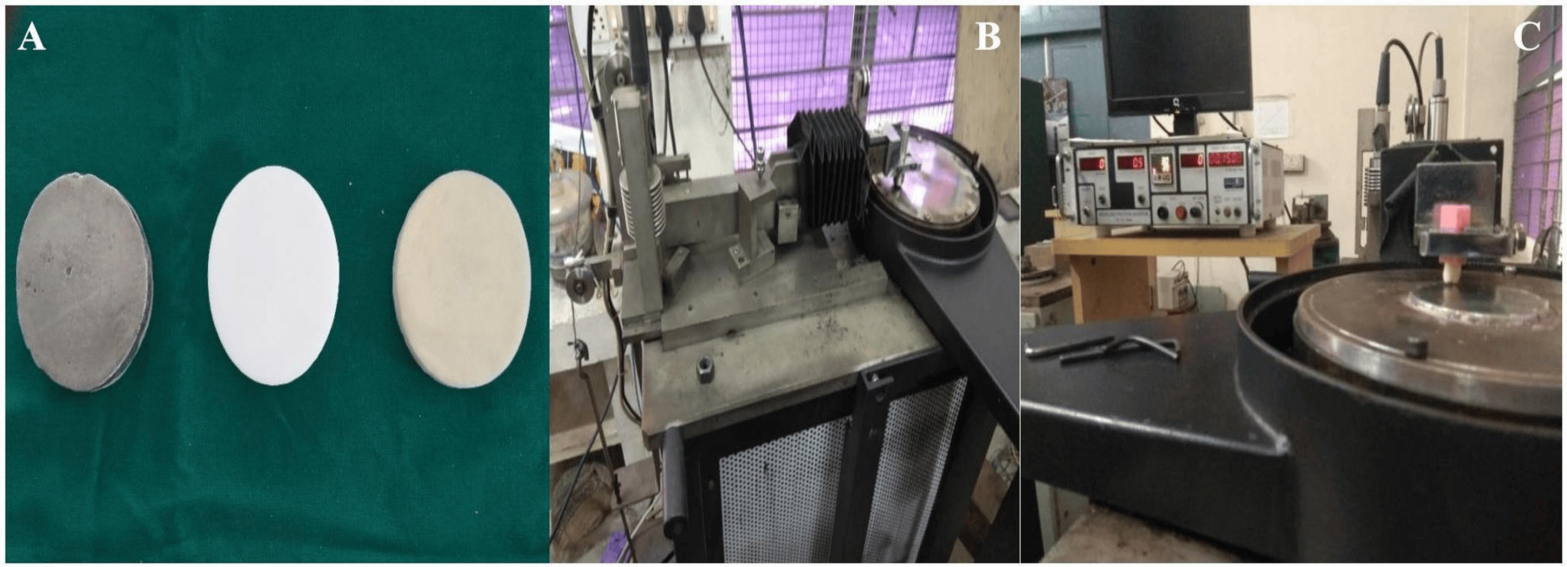
Wear testing setup A) Metal, zirconium, and metal-ceramic discs with 60 mm diameter and 10 mm thickness. B) Pin-on-disc wear testing machine. C) Crown samples attached to upper component and metal disc attached to lower component of wear testing machine.

Three types of antagonist discs measuring 60 mm in diameter and 10 mm in thickness were fabricated. Metal discs were cast from nickel-chromium alloy. Metal-ceramic discs consisted of a nickel-chromium substructure veneered with a 1.5 mm layer of feldspathic porcelain. Zirconia discs were fabricated using CAD/computer-aided manufacturing (CAM) milling of 3Y-TZP zirconia. All discs were finished and standardized prior to wear testing (Figure 3A).

Wear testing procedure

Wear testing was performed using a pin-on-disc wear testing machine operating under a two-body wear model (Figures 3B, 3C). Each specimen was mounted perpendicular to the disc surface and subjected to a constant load of 2 kg. Each sample underwent 10 wear cycles of one-hour duration against its designated antagonist disc. Continuous irrigation with distilled water maintained at 37°C was used to simulate oral lubrication. Different track diameters were employed for each specimen to prevent overlapping of wear tracks [12]. Following wear testing, the volume of material loss was measured using a non-contact three-dimensional optical profilometer. The worn surfaces were scanned to obtain surface topography, and volumetric wear loss was calculated using dedicated surface analysis software. The instrument was calibrated prior to measurement according to the manufacturer’s guidelines, with a measurement resolution of approximately 0.01 µm to ensure accuracy and reproducibility. The obtained volumetric loss values were recorded as the wear measurements for each specimen.

Statistical analysis

All statistical analyses were performed using SPSS software (Version XX, IBM Corp., Armonk, NY, USA). Descriptive statistics including mean and standard deviation were calculated for wear values in each group. The normality of data distribution was evaluated using the Shapiro-Wilk test. Since the data followed a normal distribution (p > 0.05), one-way analysis of variance (ANOVA) was applied to compare mean wear values among the experimental groups. Post hoc multiple comparisons were performed using the Tukey honestly significant difference (HSD) test. A p-value < 0.05 was considered statistically significant.

## Results

Surface wear analysis

Surface wear values for natural teeth, metal-ceramic crowns, and zirconia crowns opposing different restorative materials are presented in Table 1. One-way ANOVA demonstrated statistically significant differences in surface wear within all three sample categories. For natural teeth, the highest mean surface wear was observed against metal-ceramic discs (177.2 ± 40.4), followed by zirconia discs (94.4 ± 61.2), whereas metal discs produced the lowest wear (54.6 ± 26.4) (F = 19.1, p < 0.0001). Metal-ceramic crowns exhibited maximum wear when opposed by metal-ceramic discs (181.3 ± 37.6), followed by zirconia discs (102.2 ± 51.9), with the least wear recorded against metal discs (82.7 ± 44.5) (F = 13.7, p < 0.0001). Similarly, zirconia crowns demonstrated the greatest surface wear against metal-ceramic discs (141.4 ± 56.7), followed by zirconia discs (101.9 ± 54.9), while metal discs resulted in the lowest wear (66.5 ± 9.3) (F = 6.8, p = 0.004). Overall, metal-ceramic opposing surfaces consistently produced higher surface wear across all sample types.

**Table 1:** Comparison of surface wear among different sample types against opposing materials *Statistically significant at p < 0.05 (one-way ANOVA).

Sample Type	Opposing Material	Surface Wear (Mean ± SD)	ANOVA F	p-Value
Natural teeth	Metal disc	54.6 ± 26.4		
	Metal-ceramic disc	177.2 ± 40.4	19.1	<0.0001*
	Zirconia disc	94.4 ± 61.2		
Metal-ceramic crowns	Metal disc	82.7 ± 44.5		
	Metal-ceramic disc	181.3 ± 37.6	13.7	<0.0001*
	Zirconia disc	102.2 ± 51.9		
Zirconia crowns	Metal disc	66.5 ± 9.3		
	Metal-ceramic disc	141.4 ± 56.7	6.8	0.004*
	Zirconia disc	101.9 ± 54.9		

The Tukey HSD post hoc analysis was performed following one-way ANOVA to determine pairwise differences among the opposing materials (metal, metal-ceramic, and zirconia) . For surface wear on natural teeth, a statistically significant difference was observed between metal and metal-ceramic opposing materials (p < 0.001) and between metal-ceramic and zirconia (p = 0.004). However, the difference between metal and zirconia was not statistically significant (p = 0.087). For surface wear on metal-ceramic crowns, the comparison showed significant differences between metal and metal-ceramic (p < 0.001) and between metal-ceramic and zirconia (p = 0.006). The difference between metal and zirconia was not statistically significant (p = 0.482). For surface wear on zirconia crowns, a significant difference was found between metal and metal-ceramic opposing materials (p = 0.009). However, the comparisons between metal and zirconia (p = 0.182) and metal-ceramic and zirconia (p = 0.141) were not statistically significant (Table 2). Overall, the results indicate that metal-ceramic opposing surfaces tend to produce greater wear compared with metal and zirconia in several comparisons, whereas metal and zirconia surfaces generally exhibited comparable wear behaviour in most groups.

**Table 2:** Tukey HSD post hoc comparison for surface wear among different types of crowns Significant at **p < 0.05. HSD: honestly significant difference.

Study Groups	Comparison Between Discs	Mean Difference	p-Value	Significance	95% CI Lower	95% CI Upper
Natural teeth	Metal vs metal-ceramic	-122.6	<0.001**	Significant	-188.4	-56.8
	Metal vs zirconia	-39.8	0.087	Not significant	-105.6	26.0
	Metal-ceramic vs zirconia	82.8	0.004**	Significant	17.0	148.6
Metal-ceramic	Metal vs metal-ceramic	-98.6	<0.001**	Significant	-162.4	-34.8
	Metal vs zirconia	-19.5	0.482	Not significant	-83.3	44.3
	Metal-ceramic vs zirconia	79.1	0.006**	Significant	15.3	142.9
Zirconia crowns	Metal vs metal-ceramic	-74.9	0.009**	Significant	-132.1	-17.7
	Metal vs zirconia	-35.4	0.182	Not significant	-92.6	21.8
	Metal-ceramic vs zirconia	39.5	0.141	Not significant	-17.7	96.7

Volumetric wear analysis

Volumetric wear measurements are summarized in Table 3. Statistically significant differences were identified among opposing materials in all groups. Natural teeth showed the highest volumetric wear against metal-ceramic discs (0.0013 ± 0.0007), followed by metal discs (0.0008 ± 0.0003), while zirconia discs produced the least wear (0.0004 ± 0.0003) (F = 9.1, p < 0.0001). In metal-ceramic crowns, maximum volumetric wear occurred against metal discs (0.0023 ± 0.0011), followed by metal-ceramic discs (0.0014 ± 0.0006), with zirconia discs demonstrating the lowest wear (0.0004 ± 0.0002) (F = 14.2, p < 0.0001). Zirconia crowns exhibited the greatest volumetric wear when opposed by metal-ceramic discs (0.0010 ± 0.0001), followed by metal discs (0.0007 ± 0.0001), whereas zirconia discs resulted in minimal wear (0.0002 ± 0.0001) (F = 8.2, p < 0.0001).

**Table 3:** Comparison of volumetric wear among different sample types against opposing materials *Statistically significant at p < 0.05 (one-way ANOVA).

Sample Type	Opposing Material	Volumetric Wear (mm³) Mean ± SD	ANOVA F	p-Value
Natural teeth	Metal disc	0.0008 ± 0.0003		
	Metal-ceramic disc	0.0013 ± 0.0007	9.1	<0.0001*
	Zirconia disc	0.0004 ± 0.0003		
Metal-ceramic crowns	Metal disc	0.0023 ± 0.0011		
	Metal-ceramic disc	0.0014 ± 0.0006	14.2	<0.0001*
	Zirconia disc	0.0004 ± 0.0002		
Zirconia crowns	Metal disc	0.0007 ± 0.0001		
	Metal-ceramic disc	0.0010 ± 0.0001	8.2	<0.0001*
	Zirconia disc	0.0002 ± 0.0001		

Across all groups, zirconia opposing surfaces consistently demonstrated the lowest volumetric wear. For natural teeth, statistically significant differences were observed among all disc comparisons. The comparison between metal and metal-ceramic discs (p = 0.002), metal and zirconia discs (p = 0.011), and metal-ceramic and zirconia discs (p < 0.001) showed significant differences, indicating that the type of opposing disc material significantly influenced the volumetric wear of natural teeth.

In the metal-ceramic crown group, significant differences were found between metal and metal-ceramic discs (p = 0.003) and metal-ceramic and zirconia discs (p < 0.001). However, the comparison between metal and zirconia discs (p = 0.065) was not statistically significant, suggesting comparable volumetric wear produced by these two disc materials against metal-ceramic crowns. For zirconia crowns, significant differences were observed between metal and metal-ceramic discs (p = 0.012) and metal-ceramic and zirconia discs (p = 0.004). In contrast, the comparison between metal and zirconia discs (p = 0.118) was not statistically significant, indicating similar wear behaviour between these two disc materials when opposed to zirconia crowns (Table 4). Overall, the results indicate that metal-ceramic discs produced significantly different volumetric wear compared with the other disc materials in most comparisons, while metal and zirconia discs showed similar wear characteristics in certain groups where the differences were not statistically significant.

**Table 4:** Tukey HSD post hoc comparison for volumetric wear among different types of crowns Significant at *p < 0.05 and **p < 0.01. HSD: honestly significant difference.

Study Groups	Comparison Between Discs	Mean Difference	p-Value	Significance	95% CI Upper	95% CI Lower
Natural teeth	Metal vs metal-ceramic	-0.0005	0.002**	Significant	-0.0002	-0.0008
	Metal vs zirconia	0.0004	0.011*	Significant	0.0007	0.0001
	Metal-ceramic vs zirconia	0.0009	<0.001**	Significant	0.0012	0.0006
Metal-ceramic	Metal vs metal-ceramic	-0.0006	0.003**	Significant	-0.0003	-0.0009
	Metal vs zirconia	0.0003	0.065	Significant	0.0007	-0.0001
	Metal-ceramic vs zirconia	0.0009	<0.001**	Significant	0.0013	0.0005
Zirconia crowns	Metal vs metal-ceramic	-0.0004	0.012*	Significant	-0.0001	-0.0007
	Metal vs zirconia	0.0002	0.118	Not significant	0.0006	-0.0002
	Metal-ceramic vs zirconia	0.0006	0.004**	Significant	0.0010	0.0002

## Discussion

The concept of biomimetics has increasingly influenced restorative dentistry, with modern materials aiming to replicate the mechanical and tribological behaviour of natural enamel. Surface wear of teeth and restorative materials occurs at an ultrastructural level where microscopic asperities interact, producing scratch and ploughing patterns on enamel surfaces [13]. The structure, crystal size, and surface hardness of restorative materials play a significant role in determining the extent of antagonistic wear.

Previous investigations by Oh et al. emphasised that enamel and ceramic wear are influenced by physical factors such as hardness, fracture toughness, porosity, crystal structure, and surface finish [14]. Ceramics may be polished or glazed, and the surface treatment directly affects antagonist wear. Although ceramics generally exhibit higher hardness than human enamel, hardness alone does not reliably predict wear behaviour [15].

In the present in vitro investigation, enamel, metal-ceramic crowns, and zirconia crowns were tested against metal, metal-ceramic, and zirconia antagonists under standardised loading conditions. The experimental design was adapted from previously reported wear simulation protocols and attempted to replicate two-body wear mechanisms observed clinically [16-18]. Distilled water was used to simulate the oral environment, acknowledging that aqueous conditions can alter ceramic surface chemistry and influence wear processes [14].

The findings demonstrated that metal-ceramic antagonists consistently produced the greatest surface and volumetric wear across most experimental groups. Natural teeth opposing metal-ceramic discs exhibited the highest wear values, followed by zirconia, while metal discs produced the least wear. Similar trends were observed for zirconia crown specimens. These observations align with earlier reports suggesting that feldspathic porcelain surfaces may cause substantial enamel abrasion when surface roughness increases due to glaze loss or microfracture [19].

Interestingly, zirconia antagonists produced significantly lower volumetric wear compared with metal-ceramic surfaces. This result supports the conclusions of Ahmadzadeh et al., who reported reduced enamel wear against zirconia compared with porcelain [20]. Zirconia’s high fracture toughness (9-10 MPa) and fine-grain structure contribute to its ability to maintain smoother surfaces during functional loading, thereby minimising abrasive interactions [21]. In contrast, feldspathic porcelains exhibit lower fracture toughness, which may promote microfracture and the generation of abrasive debris.

Although zirconia exhibits greater hardness than enamel, the present results reinforce previous evidence that hardness alone is not the primary determinant of wear [22]. Surface microstructure, roughness, and environmental factors appear to exert greater influence. Studies by Mundhe et al., Jung et al., and Talibi et al. demonstrated that polished zirconia surfaces cause less enamel wear than glazed zirconia or feldspathic porcelain [3,22,23]. In the present study, polished zirconia crowns may have contributed to the relatively lower antagonistic wear observed.

From a clinical perspective, excessive occlusal wear can alter vertical dimension, occlusal stability, and temporomandibular function. Therefore, selecting restorative materials with wear characteristics compatible with natural enamel is essential [24,25]. While metal surfaces demonstrated the least wear in this study, their aesthetic limitations restrict anterior applications. Zirconia appears to provide a favourable balance between aesthetics and reduced antagonistic wear.

While clinical studies provide valuable information regarding restorative wear, they are often limited by variability in patient-specific factors, occlusal patterns, parafunctional habits, and the high cost and duration of long-term observation [26]. The results support previous findings that porcelain surfaces may contribute to increased enamel wear, whereas polished zirconia demonstrates more enamel-friendly behaviour [25]. Careful finishing and polishing of ceramic restorations are critical to minimising antagonistic wear. Future studies should incorporate larger sample sizes, advanced surface characterisation techniques such as scanning electron microscopy and three-dimensional profilometry, and experimental models that better simulate intraoral biomechanics.

In addition to the findings, the present investigation has several methodological strengths. The study was conducted under a controlled in vitro experimental environment, which allowed standardisation of variables such as load, contact time, lubrication, and antagonist material, thereby reducing confounding influences commonly encountered in clinical studies. A standardised wear simulation protocol using a pin-on-disc two-body wear model was employed to reproduce clinically relevant occlusal wear mechanisms. Furthermore, the inclusion of multiple restorative material combinations (natural enamel, metal-ceramic, and zirconia) opposing different antagonist surfaces enabled a comprehensive comparison of wear behaviour across commonly used restorative systems. The study also incorporated both surface and volumetric wear assessments, providing a more detailed evaluation of material loss and antagonist interaction. These methodological considerations enhance the reliability of the experimental outcomes and contribute to a clearer understanding of the tribological performance of restorative materials.

However, several limitations should be acknowledged when interpreting the results. First, each experimental subgroup consisted of a relatively small sample size (n = 10), which may limit the statistical power and generalisability of the findings. Second, the study employed a two-body wear model, whereas intraoral wear frequently involves three-body wear mechanisms mediated by food particles, saliva components, and debris, which may influence the wear patterns observed clinically. Third, the experimental design did not incorporate long-term cyclic fatigue or thermal fluctuations, both of which are important factors influencing restorative material degradation in the oral environment. Furthermore, although surface finishing and polishing protocols were standardised, variations in surface roughness after clinical adjustment or glazing loss could alter antagonist wear behaviour in real clinical settings. Therefore, the findings should be interpreted within the context of these experimental constraints. Future studies incorporating larger sample sizes, long-term fatigue simulation, thermocycling, and three-body wear models may provide further insight into the clinical performance of restorative materials.

## Conclusions

Wear of teeth and restorative materials is a multifactorial phenomenon governed by mechanical, chemical, and biologic interactions within the oral environment. Within the limitations of this controlled in vitro experimental study, significant differences in wear behaviour were observed among natural enamel, metal-ceramic crowns, and zirconia crowns when opposed by different restorative surfaces. Metal-ceramic antagonists produced the highest wear values across most tested groups, whereas nickel-chromium metal surfaces demonstrated the least wear. Zirconia exhibited intermediate wear characteristics and produced substantially lower antagonistic wear compared with metal-ceramic surfaces. These findings suggest that hardness alone is not a reliable predictor of wear and that surface microstructure, fracture behaviour, and material properties play an important role in wear mechanisms.

Although zirconia demonstrated favourable wear compatibility compared with feldspathic porcelain surfaces under the experimental conditions, these findings should be interpreted cautiously, as the study was conducted in a laboratory environment using a two-body wear simulation model. Intraoral conditions involve complex factors such as saliva composition, temperature variations, occlusal dynamics, and three-body wear mechanisms that may influence clinical outcomes.
